# A screening method for phosphohistidine phosphatase 1 activity

**DOI:** 10.3109/03009734.2011.585253

**Published:** 2011-06-29

**Authors:** Ulla Beckman-Sundh, Bo Ek, Örjan Zetterqvist, Pia Ek

**Affiliations:** ^1^Department of Medical Biochemistry and Microbiology, Uppsala University, Uppsala, Sweden; ^2^Toxicology Division, National Food Administration, Uppsala, Sweden; ^3^Department of Physical and Analytical Chemistry, Uppsala University, Uppsala, Sweden; ^4^Science for Life Laboratory, Department of Medical Biochemistry and Microbiology, Uppsala University, Uppsala, Sweden

**Keywords:** Activity, assay, phosphohistidine phosphatase, PHPT1, protein histidine phosphatase

## Abstract

**Introduction:**

Research in the field of protein-bound phosphohistidine phosphorylation has been hampered by the difficulties in analysis and detection of phosphohistidine. Therefore a screening method was developed primarily for the analysis of phosphohistidine phosphatase 1 (PHPT1) activity.

**Methods:**

A highly positively charged substrate, Ac-Val-Arg-Leu-Lys-His-Arg-Lys-Leu-Arg-pNA, containing the peptide surrounding the phosphorylated histidine in ion channel KCa3.1 was chemically phosphorylated using phosphoramidate. Excess phosphoramidate was removed by anion exchange chromatography using a micro spin column. After incubation of the eluate with PHPT1, the removed phosphate was bound on a consecutive anion exchange spin column. The eluate was assayed in a micro plate format for remaining phosphate in the substrate Ac-Val-Arg-Leu-Lys-His(P)-Arg-Lys-Leu-Arg-pNA. Histone H4, also highly positive in charge, was subjected to the same procedure to explore the possibility to use other substrates to PHPT1 in this assay format.

**Results:**

It was found that Ac-Val-Arg-Leu-Lys-His(P)-Arg-Lys-Leu-Arg-pNA and phosphohistone H4 were dephosphorylated by PHPT1. The apparent K_m_ for Ac-Val-Arg-Leu-Lys-His(P)-Arg-Lys-Leu-Arg-pNA was in the order of 10 μM.Using this method, phosphohistidine phosphatase activity was detected in mouse liver cell sap with Ac-Val-Arg-Leu-Lys-His(P)-Arg-Lys-Leu-Arg-pNA as substrate.

**Discussion:**

The described method for determination of PHPT1 activity is comparably much easier and faster than presently used methods for detection of phosphohistidine phosphatase activity. It is also sensitive, since the lower activity limit was 5 pmol phosphate released per min. It has the potential to be used both for more rapid screening for inhibitors and activators to phosphohistidine phosphatases and for screening of histidine kinases.

## Introduction

The formation and degradation of protein-bound phosphohistidine has met with increased interest during recent time (see Klumpp et al. (1) and references therein). Instrumental in this development has been the discovery, independently by two groups, of a mammalian phosphohistidine phosphatase (PHPT1), also referred to as protein histidine phosphatase (2,3). It seems to be ubiquitously expressed, with an especially high expression on dividing epithelial cells (4).

Essential issues in the current studies on PHPT1 are the identification of its natural substrates and the development of convenient methods for its assay. One problem connected with these tasks is due to the well known lability of phosphohistidine at acid conditions, which may also partly explain the rather slow pace of phosphohistidine-related research, despite the early discovery of protein-bound phosphohistidine by Boyer et al. in 1962 (5). Another problem in developing assays for PHPT1 is that the substrates identified so far are either dephosphorylated at low rates or require quite laborious assay procedures, or both. Therefore, a simpler assay was attempted, as described in the present work.

The standard substrate for PHPT1 used so far by us is the peptide succinyl-Ala-His(P)-Pro-Phe-*p*-nitroanilide, where a comparatively time-consuming HPLC is required for the separation of phosphorylated and dephosphorylated peptide (2). With this assay, using 7 μM phosphopeptide, the pure pig liver PHPT1 displayed an activity of 3 μmol·min^-1^·mg^-1^, and the human recombinant PHPT1 9 μmol·min^-1^·mg^-1^. With 1 mM phosphoramidate as substrate, the rate was 0.6 μmol·min^-1^·mg^-1^ for the pig liver enzyme.

In their original work on rabbit liver PHPT1, Klumpp et al. (3) used the bacterial autophosphorylated chemotaxis protein A (CheA) as substrate, at a concentration of 5 μM, which gave a specific activity of the pure enzyme of only about 12 pmol·min^-1^·mg^-1^, i.e. at least five orders of magnitude lower than the values obtained by Ek et al. (2).

Later, Klumpp and co-workers identified phosphorylated ATP citrate lyase (ACL) and β-subunit of G-protein (Gβ) as substrates of PHPT1 (6,7). Gβ is phosphorylated by the action of NDPK-B, as detailed in Wieland et al. (8). The Mg^2+^-dependent phosphorylation of purified ACL occurs either by autophosphorylation or by nucleoside diphosphate kinase A (NDPK-A), while ACL in a crude rat liver extract can be phosphorylated also in the presence of EDTA (cf. Wagner (9)). We would suggest that the phosphorylation of ACL in EDTA-containing crude extracts may be explained by the phosphoryl transfer from NDPK-A, which can be autophosphorylated also in EDTA, although at a considerably lower rate than that obtained at excess of Mg^2+^ (cf. Wålinder et al. (10)). The analyses of phosphorylated substrates were in all three cases SDS-PAGE followed by autoradiography after phosphorylation with radiolabelled ATP. No specific activities of PHPT1 for these two substrates are reported, but the rate of dephosphorylation appears to be quite low. A detailed description of their methods and the interplay between NDPK and PHPT1 were given by Wieland et al. (8).

In a joint project with the group of Kowluru the Klumpp group has also indicated a role for PHPT1 in insulin secretion in pancreatic β-cells (11), and together with Attwood and Besant and co-workers they have, using NMR, shown a dephosphorylation by PHPT1 of phosphoramidate and of chemically phosphorylated peptides representing the amino acid sequences around the two histidine residues (His-18 and His-75) of histone H4 (12). The estimated rates of dephosphorylation seem to be of the same order as reported for phosphoramidate and Suc-Ala-His(P)-Pro-Phe-pNA by Ek et al. (2).

The clearest demonstration so far of a physiological role of PHPT1 in regulatory protein phosphorylation has been reported by the group of Skolnik which has shown that the potassium channel KCa3.1 in human CD4 T lymphocytes is activated in a NDPK-B-dependent phosphorylation (13) and is inactivated by the action of PHPT1 (14). The phosphorylation of KCa3.1 takes place at histidine 358. This residue is located in the carboxyl terminus of KCa3.1 and is surrounded by several basic residues, in the sequence Val-Arg-Leu-Lys-His-Arg-Lys-Leu-Arg.

Given this background, we chose to make use of the highly basic nature of this sequence and tested the chemically phosphorylated peptide acetyl-Val-Arg-Leu-His(P)-Arg-Lys-Leu-Arg-para-nitroanilide (Ac-Val-Arg-Leu-Lys-His(P)-Arg-Lys-Leu-Arg-pNA) as a substrate for PHPT1. The addition of the N-terminal acetyl group was aimed to prevent an attack of aminopeptidases in crude cell extracts, and the C-terminal para-nitroanilide group was both aimed to prevent the action of carboxypeptidases and to facilitate a sensitive and quantitative tracing of the substrate. Phosphorylation was achieved chemically by incubation with phosphoramidate. The phosphate in the product after incubation with enzyme was measured by a malachite method after removal of excess phosphoramidate and released phosphate on DEAE-Sephacel micro spin columns.

Recently, Attwood et al. reported that PHPT1 appeared unable to dephosphorylate histone H4 that had been phosphorylated with a thymus histidine kinase (12). However, since histone H4 contains two histidine residues (His-18 and His-75) and either 1-phosphohistidine or 3-phosphohistidine can occur depending on the source of the histidine kinase (15), there may be as much as four possible ways to mono-phosphophorylate histone H4. If only one of these versions represents the product of the thymus histidine kinase, there is still a possibility that at least one of the remaining versions can be dephosphorylated by PHPT1. Assuming that the chemical phosphorylation of histone H4 by phosphoramidate can give rise to more than one variant of histidine-phosphorylated histone H4 (16,17), we applied the new assay described in this report in an investigation of the dephosphorylation by PHPT1 of chemically phosphorylated histone H4, also highly positive in charge. This was also performed in order to investigate if the method can be used for other positively charged phosphohistidineproteins of interest instead of the peptide in future research in the field of phosphorylation-dephosphorylation of histidine.

## Materials and methods

### Materials

DEAE-Sephacel and Sephadex G-50 were from GE-Health Care, Sweden. Micro Bio-Spin columns were obtained from BioRad, USA. Ac-Val-Arg-Leu-Lys-His-Arg-Lys-Leu-Arg-pNA and Ac-Val-Arg-Leu-Lys-Ala-Arg-Lys-Leu-Arg-pNA were synthesized by Genscript, USA by order. Malachite green reagent was from Bie-Berntsen, Denmark. Antibodies raised in rabbit against histone H4 were from Upstate, USA. Secondary polyclonal swine anti-rabbit antibodies, conjugated with alkaline phosphatase, were from Dako, Denmark and 5-bromo-4-chloro-3-indolyl phosphate (BCIP) was from Sigma, USA. Non-radioactive phosphoramidate was synthesized by applying the method described for [^32^P]phosphoramidate by Buckler and Stock (18). It was lyophilized and stored at -20°C until use, when it was dissolved in deionized water to a concentration of 0.2 M. Solutions of phosphoramidate were then stored at -80°C in aliquots that were thawed only once before use. Human recombinant phosphohistidine phosphatase (PHPT1) was expressed and purified as described by Ma et al. (19). Human recombinant histone H4 was from New England Biolabs, UK. The recombinant protein was delivered, at 1 mg/mL, in 20 mM sodium phosphate (pH 7.0), 300 mM NaCl, 1 mM EDTA, and 1 mM dithiothreitol. Before use, this solvent was changed by centrifugation of 50 μL aliquots through a 210 μL DEAE-Sephacel gel equilibrated in 10 mM HCl for 2 min at 900 *g* according to the manufacturer's instruction. Histone Type II-S, which has a high concentration of histone H4, was from Sigma and was further purified as described below. All peptides aimed at ESI-ms(-ms) were pre-purified on C18 tips obtained from Eppendorf Ltd, UK according to instructions. After elution in usually 10 μL of 60% acetonitrile/1% acetic acid, aliquots of 2 μL were transferred to spray needles (New Objective Inc., USA). Analysis was performed manually and immediately after purification in order to limit dephosphorylation in the acid solution. Analysis was on a LTQ-ft Ultra (Thermo Scientific, Germany). Spectra were further analysed in the mMass software (20).

### Methods

MALDI-TOF mass spectrometry was performed by Dr Åke Engström at the Department of Medical Biochemistry and Microbiology. UV-absorption was measured by use of a NanoDrop2000c Spectrophotometer from Thermo Scientific. Mouse liver cytosol was prepared by the small-scale method, as described for pig liver cytosol (2), except that 1 mM Pefabloc was used instead of diisopropyl fluorophosphate as a protease inhibitor.

#### Purification of histone H4

Calf thymus histone Type II-S (Sigma), 0.5 mL of a 40 mg/mL solution in 8 M urea/10 mM HCl, was chromatographed on a 20 mL (0.5 × 25 cm) Sephadex G-50, equilibrated, and eluted with 10 mM HCl. Fractions were analysed by SDS-PAGE and Western blot as described below, and peak fractions containing histone H4 were used for further experiments. The pooled material was diluted in 10 mM HCl, to a histone H4 concentration of 1 mg/mL and was kept at -20°C until use.

#### Equilibration of DEAE-Sephacel

DEAE-Sephacel was left to sediment, decanted and equilibrated in 25 mM Tris/HCl, pH 8.0 for peptides and pH 8.5 for histone H4. The final suspension was 60% (v/v). Micro Bio-Spin columns were packed with a predetermined volume of the suspension that depended on the volume of the sample (generally 700 μL for samples of 400–525 μL and 350 μL for samples of 50–250 μL) and centrifuged for 10 s at 900 *g*. Then 500 μL of the equilibration buffer was added to the columns followed by centrifugation for 1 min at 900 *g*.

#### Histidine phosphorylation of peptides and histone H4

Aliquots of 25 μL of peptide solutions (generally 1 mM) were mixed with 25 μL 25 mM Tris/HCl, pH 8.0, and incubated with 1 μL 0.2 M phosphoramidate at room temperature for the indicated times. Similarly, 25-μL aliquots of a solution of histone H4 (1 mg/mL, 88 μM) in 10 mM HCl were mixed with 25 mM Tris/HCl, pH 8.5 to give pH 7.8, and incubated with 1 μL 0.2 M phosphoramidate for 48 h at room temperature.The incubation was interrupted by freezing at -80°C or, if immediately used, by centrifugation on 200 μL DEAE-Sephacel Micro Bio-Spin columns for 2 min at 900 *g*. Tween 20 was added to a final concentration of 0.02% (v/v) before the centrifugation step to improve the recovery of phosphohistone. The eluted volume was centrifuged on a second gel after another addition of Tween to 0.02%, and the eluate was used for further experiments. The volume of the samples increased by 2%–4% during these procedures; a sample of 50 μL increased to 52–54 μL after centrifugation. This general procedure using the Micro Bio-Spin columns could also be adapted for larger incubation volumes.

#### Dephosphorylation of phosphorylated peptides and histone H4

Aliquots of 50 μL of the eluates from the Micro Bio-Spin columns were incubated at 30°C with 1 μL of PHPT1 or test samples at indicated concentrations, for varied times. Tween 20 was added to a final concentration of 0.02% and was present during the dephosphorylation. The incubations were interrupted by centrifugation for 2 min at 900 *g* on 210 μL DEAE-Sephacel Micro Bio-Spin columns. The eluate was analysed for acid-labile phosphate as described below. The absorbance at 320 nm and a molar absorbance of 6050 M^-1^·cm^-1^ was used to determine the recovery of phosphorylated and unphosphorylated peptides. The recovery of total histone H4 was determined by measuring the absorbance at 280 nm, using the A_280_ value 0.524 for 1 mg/mL and a light-path of 1 cm.

#### Phosphate analysis

The eluate was analysed for phosphate using malachite green reagent. The acid milieu of this reagent (1 M HCl) permitted the analysis of acid-labile, N-linked phosphate. Thus, a sample of 50 μL was incubated with 50 μL of the malachite green reagent for minimally 2 h at room temperature in micro plates. This time was sufficient to release all of the acid-labile phosphate as orthophosphate. The absorbance was measured at 620 nm, and the amount of phosphate was calculated using standards from 0 to 2 nmol phosphate in 50 μL H_2_O where each pmole phosphate gave an absorbance of 0.0002, so the lower sensitivity limit was in the order of 2–5 pmoles/min under the condition used. All buffers used in this study were tested as diluents of the standards, with the same results. The coefficients of correlation for the standard curves were always above 0.99.

#### SDS-PAGE

SDS-PAGE was performed in 12% polyacrylamide gels in accordance with standard protocols, and the gels were stained with Coomassie brilliant blue.

#### Western blot

The proteins in an unstained SDS-PAGE gel were transferred to a nitrocellulose membrane using a semi-dry transfer cell from BioRad according to their instructions. Coating was performed in a buffer containing 5% (w/v) dry milk, and the dilution of anti-histone H4 antibodies was 1/10,000. An alkaline phosphatase-conjugated anti-rabbit IgG and BCIP were used for detection of histone H4 according to the instructions from the manufacturer.

## Results and discussion

### Phosphorylation of peptides and histone H4 by phosphoramidate

**Figure 1.**
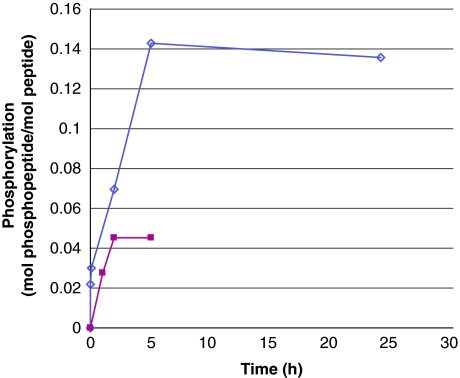
Phosphorylation of peptides by phosphoramidate as a function of time. The concentration of Ac-Val-Arg-Leu-Lys-His-Arg-Lys-Leu-Arg-pNA and Ac-Val-Arg-Leu-Lys-Ala-Arg-Lys-Leu-Arg-pNA was 0.5 mM and that of phosphoramidate 3.9 mM. The incubation was performed at room temperature and interrupted by centrifugation of 50 μL of the reaction mixture through two consecutive spin columns containing 210 μL DEAE-Sephacel equilibrated in 25 mM Tris/HCl, pH 8.0. The phosphate and peptide concentration in the final eluate was determined by malachite reagent and by measuring the absorbance at 320 nm, respectively. The values are given as means of duplicate analysis. (◊) Ac-Val-Arg-Leu-Lys-His-Arg-Lys-Leu-Arg-pNA and (▪) Ac-Val-Arg-Leu-Lys-Ala-Arg-Lys-Leu-Arg-pNA. Details are given in Material and methods.

As mentioned in the Introduction, we first chose to work with a peptide representing the site around the phosphorylatable histidine 358 in the calcium-stimulated potassium channel KCa3.1 (14). From the progress of the phosphorylation of 0.5 mM Ac-Val-Arg-Leu-Lys-His-Arg-Lys-Leu-Arg-pNA (Figure 1), it is concluded that an incubation time of more than 24 h would not significantly increase the phosphorylation level above the generally obtained 0.15 mol phosphate per mol of peptide. Therefore, this incubation time was chosen for standard preparations. The about 3-fold lower level of histidine phosphorylation, compared to what may be expected from the peptide phosphorylation by phosphoramidate described by Attwood et al. (12), may be partly explained by the 10- and 20-fold lower initial concentrations of the basic peptide and phosphoramidate, respectively, in our experiments, which should also give lower rates of phosphorylation. In addition, the phosphorylated peptide is labile in itself at pH 8.0, which may further decrease the net rate of the chemical phosphorylation. Finally, due to the lability of phosphoramidate (12,18), its concentration is gradually decreased during the incubation. Still, the low initial concentration of phosphoramidate (3.9 mM) was a deliberate choice in order not to exceed the binding capacity of the DEAE-Sephacel columns used (see below).

Under the same conditions, the control peptide Ac-Val-Arg-Leu-Lys-Ala-Arg-Lys-Leu-Arg-pNA displayed a background phosphorylation of 0.05 mol/s/mol enzyme, despite its lack of histidine. The nature of this phosphorylation and the phosphorylation ratio between the two were determined using mass spectrometry. Several forms (i.e. charges) of the peptide were found. For the His-containing peptide two differently charged phosphorylated variants were seen (+3 and +4). The nature of the phosphohistidine isomer in Ac-Val-Arg-Leu-Lys-His(P)-Arg-Lys-Leu-Arg-pNA was not determined, but it was considered to be 3-phosphohistidine, rather than 1-phosphohistidine, due to the 24 h phosphorylation time used (12,21,22). By using both ion-trap for fragmentation and analysis it was possible to detect a complete y-series for the phosphorylated peptide (not shown). When ion-cyclotron analysis was attempted, only partial series were obtained even in the case fragmentation was by electron-capture dissociation (ECD).

For the Ala-peptide an almost complete y-series was seen indicating that the Lys in position 7 was phosphorylated. Scrutinizing the spectra, a short stretch of the b-series could also be seen (b4, b5, and b6), and this showed that also the Lys in position 4 was phosphorylated. No doubly phosphorylated peptides were seen, but the reason for this could be difficulties in analysing it. Internal fragments could not be used since the peptide unfortunately contains a mirror plane assuming that Ile and Leu cannot be distinguished by the mass spectrometry. In the Ala-peptide a peak corresponding to the immonium ion of phospholysine was detected. This peak was also seen in the His-peptide, and this supports the use of the Ala-peptide as control peptide. Thus both lysines could have been modified which also is in accordance with results obtained by Kowalewska et al. (23). The ratio in phosphorylation degree as determined chemically between the two was confirmed by mass spectrometry. It should be mentioned that PHPT1 dephosphorylated Ac-Val-Arg-Leu-Lys(P)-Ala-Arg-Lys(P)-Leu-Arg-pNA at about the same initial rate as that of the background release of phosphate at pH 8.0 from the phosphorylated Ala- or His-containing peptide, i.e. 2–5 pmol/min.

The chemical phosphorylation of 0.09 mM histone H4 was performed at the same low concentration of phosphoramidate (3.9 mM) as used for the peptides. The buffer, in which the recombinant histone H4 was delivered, was shown to give variable and high phosphate backgrounds, probably since its high ionic concentration may have approached the binding capacity of the DEAE-Sephacel column. This problem was circumvented by changing the solvent before phosphorylation using micro spin columns equilibrated in 10 mM HCl. When mixed with the incubation buffer, a pH of 7.8 was obtained for both the purified and the recombinant form. The phosphorylation was extended to 48 h in order to obtain the highest possible phosphorylation (data not given). The chromatography on the DEAE-Sephacel micro spin column after the phosphorylation was performed at pH 8.5 instead of pH 8.0 in order to increase the stability of both phosphoramidate and the phosphohistone product. By using this protocol, the calf thymus histone H4 purified from Sigma Histone Type II-S was phosphorylated to about 0.5 mol/mol as was the human recombinant histone H4. The losses of histone during centrifugation through each DEAE-Sephacel column were in the range of 20% for both histone H4 forms. Therefore a more exact figure could not be given.

Under the conditions used for isolation of phosphopeptides and phosphohistone H4, the phosphoramidate and any released orthophosphate were completely bound to the DEAE-Sephacel. This was ascertained after doubling the phosphoramidate amount and measuring the sum of acid-labile phosphate and orthophosphate in the eluate from two consecutive DEAE-Sephacel columns (420 μL followed by 210 μL gel). Furthermore, no phosphate was detected in the eluates at zero time of phosphorylation, which provides proof that neither phosphoramidate nor any released orthophosphate stayed bound to the basic peptides or to histone H4 during the passage through the DEAE-Sephacel columns.

### Dephosphorylation of Ac-Val-Arg-Leu-Lys-His(P)-Arg-Lys-Leu-Arg-pNA

**Figure 2.**
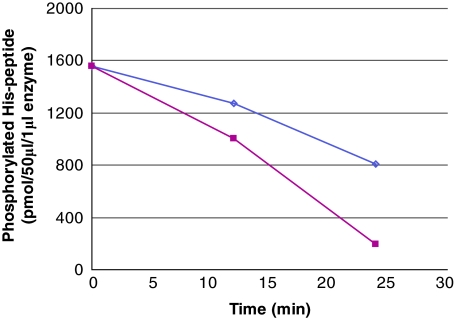
The PHPT1 activity as a function of enzyme concentration. The concentration of Ac-Val-Arg-Leu-Lys-His(P)-Arg-Lys-Leu-Arg-pNA was 24 μM in this experiment, and the enzyme was diluted 1/10 (▪) and 1/20 (◊), the former corresponding to 2.5 pmol per 50 μL incubation. The dephosphorylation was performed at 30°C and was interrupted by centrifugation of 50 μL of the reaction mixture through a spin column containing 210 μL DEAE-Sephacel equilibrated in 25 mM Tris/HCl, pH 8.0. The phosphate in the final eluate was determined by malachite reagent and peptide by absorbance at 320 nm. Details are given in Material and methods.

The dephosphorylation by recombinant PHPT1 of 24 μM phosphopeptide, in the presence of 136 μM unphosphorylated peptide (phosphorylation degree around 0.15 mol/mol), is described in Figure 2. The net rate, as measured for the first 10 minutes, was essentially proportional to the PHPT1concentration and amounted to about 0.2 mol per second per mol PHPT1.

That the dephosphorylation was essentially a dephosphorylation of phosphohistidine was supported by the fact that the phosphate bound to Ac-Val-Arg-Leu-Lys-Ala-Arg-Lys-Leu-Arg-pNA (0.05 mol/mol) was not detectably sensitive to PHPT1. In control assays with no enzyme added a slow release of phosphate, 4 pmol/min (mean of three analyses), was seen for both peptides. A dephosphorylation activity below 5 pmol/min is uncertain due to the sensitivity of the phosphate analysis (0.0002 absorbance units/pmol) under the conditions used.

**Figure 3.**
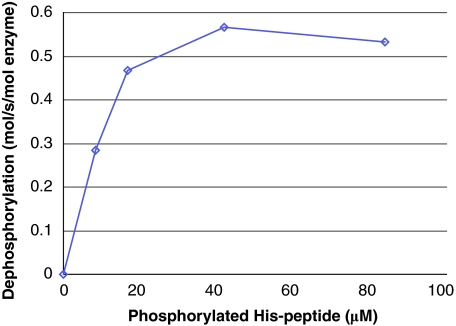
Activity of PHPT1 as a function of phosphopeptide concentration. After phosphorylation of 2 mM Ac-Val-Arg-Leu-Lys-His-Arg-Lys-Leu-Arg-pNA, phosphoramidate was removed by centrifugation through DEAE-Sephacel as described in Material and methods and diluted. The concentration of phosphopeptide was determined in each sample. Purified PHPT1 (1 pmol) was added to each concentration, and dephosphorylation was performed at 30°C in duplicate for 4, 8, and 12 min for each concentration. The reaction was interrupted by centrifugation of 50 μL through a spin column containing 210 μL DEAE-Sephacel equilibrated in 25 mM Tris/HCl, pH 8.0. The phosphate in the final eluate was determined by malachite reagent and peptide by absorbance at 320 nm. Details are given in Material and methods. The initial rate for each phosphopeptide concentration was plotted as a function of phosphopeptide concentration. The experiment was repeated three times with similar results.

The dependence of the PHPT1 activity on the phosphopeptide concentration is shown in Figure 3. An apparent K_m_ was estimated to about 10 μM Ac-Val-Arg-Leu-Lys-His(P)-Arg-Lys-Leu-Arg-pNA. No K_m_ is given in earlier works to compare with, but the activity towards the peptide used in the present work at least is in the same range as the activities for the peptides derived from histone H4 described by Attwood et al. (12). We compared in some experiments the peptide substrate Suc-Ala-His(P)-Pro-Phe-pNA (24 μM) used in Ek et al. (2) to Ac-Val-Arg-Leu-Lys-His(P)-Arg-Lys-Leu-Arg-pNA (8 μM). Both gave similar results using human recombinant PHPT1 as enzyme (data not given).

It should be borne in mind that there was 85% product present during the dephosphorylation reaction. No product inhibition could be seen at a total concentration of 0.6 mM of phosphorylated and unphosphorylated peptide.

For larger sets of experiments during a limited time it is worth purifying the phosphopeptide from the mixture by ion exchange chromatography. We deliberately describe herein experiments that can easily be performed on a more on and off basis, thereby avoiding the problem with slow spontaneous dephosphorylation.

### PHPT1 activity in liver cell sap

When cytosol from mouse liver prepared as described in Ek et al. (2) was assayed for PHPT1 activity using 30 μM Ac-Val-Arg-Leu-Lys-His(P)-Arg-Lys-Leu-Arg-pNA (together with 170 μM unphosphorylated peptide) as substrate, the activity was 2.4 μmol·min^-1^ per mg of cytosol protein. This is similar to that found earlier in porcine liver cytosol using 7 μM Suc-Ala-His(P)-Pro-Phe-pNA as the substrate (2). It must, however, be emphasized that the activity obtained varied, probably due to the delicate balance between phosphohistidine phosphorylation and dephosphorylation. The purification procedure is more rapid when small liver samples are used, which means that ATP can still be present, thereby influencing this balance. No degraded phosphopeptide, that might have occurred during the incubation with mouse liver cytosol, could be detected by mass spectrometry analysis (MALDI-ms) in a sample where the Ac-Val-Arg-Leu-Lys-His(P)-Arg-Lys-Leu-Arg-pNA was dephosphorylated by 50%. The present method may therefore be applicable to a screening for PHPT1 in crude extracts.

### Dephosphorylation of chemically phosphorylated histone H4

**Figure 4.**
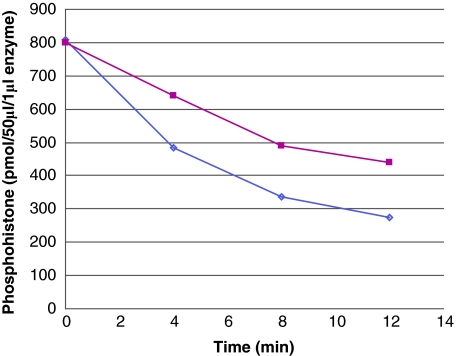
Dephosphorylation of purified and recombinant histone H4. Histone H4 was, after phosphorylation with phosphoramidate to 1.29 mol/mol, dephosphorylated with 2 pmoles PHPT1 for indicated times. The reaction was performed at 30°C and was interrupted by centrifugation of 50 μL of the reaction mixture through a spin column containing 210 μL DEAE-Sephacel equilibrated in 25 mM Tris/HCl, pH 8.5 at indicated times. Each time point was analysed in duplicate. The phosphate in the final eluate was determined by malachite reagent and histone by absorbance at 280 nm. Details are given in Material and methods.(◊) 40 μM purified phosphohistone H4, and (▪) 20 μM recombinant phosphohistone H4.

Human recombinant histone H4 and calf thymus histone H4 purified from histone Type II-S were, after phosphorylation with phosphoramidate, used as substrates for human recombinant PHPT1. The result of one experiment is shown in Figure 4. As measured at 15.7 μM phosphohistone in both cases, the rate of dephosphorylation of phosphorylated, purified histone H4 was 0.6 mol·sec^-1^ per mol PHPT1, while that of the phosphorylated recombinant histone H4 was 0.3 mol·sec^-1^ per mol. The difference is further underlined by the fact that the dephosphorylation rate for the phosphorylated, purified H4 was higher despite its 2-fold lower phosphorylation degree. The analyses were performed twice, so these differences are probably not statistically significant since the phosphorylation and dephosphorylation varied in the order of 5% between different experiments. A difference in the relative phosphorylation of His-18 and His-75 of either histone H4 or a presence of an interfering post-translational modification in the purified calf thymus histone H4 may, however, be considered.

Irrespective of the differences, the rates obtained are remarkably high and are comparable to the rates obtained for PHPT1 with the basic peptide in this work as well as with the Suc-Ala-His(P)-Pro-Phe-pNA used in a previous work by Ek et al. (2) and histone H4-derived phosphopeptides studied by Attwood et al. (12). This appears to be, by far, the highest activity reported so far of a dephosphorylation by PHPT1 of a histidine-phosphorylated protein and promotes the possibility of using the method for other highly positively charged molecules.

To the extent that the chemical phosphorylation of histone H4 by phosphoramidate gives rise to phosphohistidines that are representative also of an *in-vivo* phosphorylation of histone H4, the high rates of dephosphorylation by PHPT1 reported here are of significant interest and open the possibility that at least one type of phosphohistidine in histone H4 may be a substrate for PHPT1 also *in vivo*.

## Conclusion

This paper has described a new sensitive method for the analysis of phosphohistidine phosphatase (PHPT1). An essential advantage of the method is its simplicity in both the preparation of the phosphorylated labile substrate and the actual measurement of the PHPT1-activity. The method was designed for the use with substrates that remain highly basic even after phosphorylation. Significant rates of dephosphorylation by PHPT1 were displayed for chemically phosphorylated histone H4 and a phosphopeptide representing a phosphohistidine site of the potassium channel KCa3.1. Although not the subject of the present work, we venture to suggest that the preparative part of the method may be useful also in a search for enzymatic phosphorylation of histone H4 and basic, histidine-containing peptides, without a need for radioactively labelled phosphate donors, since the only limiting step for large-scale analysis is the centrifuge(s) needed. The method can also facilitate the search for inhibitors and activators to phosphohistidine phosphatases like PHPT1. Efficient inhibitors would increase the sensitivity of the detection of the histidine phosphorylating agents in crude cell systems.
